# Characterizing the sarcoplasmic proteome of aged pork chops classified by purge loss

**DOI:** 10.1093/jas/skad046

**Published:** 2023-02-08

**Authors:** Logan G Johnson, Chaoyu Zhai, Leah M Reever, Kenneth J Prusa, Mahesh N Nair, Elisabeth Huff-Lonergan, Steven M Lonergan

**Affiliations:** Department of Animal Science, Iowa State University, Ames, Iowa 50011, USA; Department of Animal Science, University of Connecticut, Storrs, Connecticut 06269-4040, USA; Department of Food Science and Human Nutrition, Iowa State University, Ames, Iowa 50011, USA; Department of Food Science and Human Nutrition, Iowa State University, Ames, Iowa 50011, USA; Department of Animal Sciences, Colorado State University, Fort Collins, Colorado 80523, USA; Department of Animal Science, Iowa State University, Ames, Iowa 50011, USA; Department of Animal Science, Iowa State University, Ames, Iowa 50011, USA

**Keywords:** bottom-up proteomics, liquid chromatography-mass spectrometry, pork, purge, tandem mass tag, water-holding capacity

## Abstract

Unpredictable variation in quality, including fresh pork water-holding capacity, remains challenging to pork processors and customers. Defining the diverse factors that influence fresh pork water-holding capacity is necessary to make progress in refining pork quality prediction methods. The objective was to utilize liquid chromatography and mass spectrometry coupled with tandem mass tag (TMT) multiplexing to evaluate the sarcoplasmic proteome of aged pork loins classified by purge loss. Fresh commercial pork loins were collected, aged 12 or 14 d postmortem, and pork quality and sensory attributes were evaluated. Chops were classified into Low (*N* = 27, average purge = 0.33%), Intermediate (*N* = 27, average purge = 0.72%), or High (*N* = 27, average purge = 1.19%) chop purge groups. Proteins soluble in a low-ionic strength buffer were extracted, digested with trypsin, labeled with 11-plex isobaric TMT reagents, and detected using a Q-Exactive Mass Spectrometer. Between the Low and High purge groups, 40 proteins were differentially (*P* < 0.05) abundant. The Low purge group had a greater abundance of proteins classified as structural and contractile, sarcoplasmic reticulum and calcium regulating, chaperone, and citric acid cycle enzymes than the High purge group. The presence of myofibrillar proteins in the aged sarcoplasmic proteome is likely due to postmortem degradation. These observations support our hypothesis that pork chops with low purge have a greater abundance of structural proteins in the soluble protein fraction. Together, these and other proteins in the aged sarcoplasmic proteome may be biomarkers of pork water-holding capacity. Additional research should establish the utility of these proteins as biomarkers early postmortem and over subsequent aging periods.

## Teaser

The fractionation and proteomic analysis of the aged sarcoplasmic proteome offers an unparalleled view of the postmortem biochemical and proteolytic events associated with pork water-holding capacity.

## Introduction

Water is the most abundant component of muscle and meat tissue and is important to provide an aqueous environment for normal muscle metabolism. The ability of meat tissue to retain moisture within and between muscle cells during postmortem storage is commonly termed water-holding capacity. Fresh meat can lose a significant portion of its weight as purge or drip loss, and this purge loss is not only water but also soluble protein (Savage et al., 1990). Purge loss reduces the weight of a product, is unappealing and undesirable for many consumers in retail packaging, and ultimately decreases the eating experience for consumers (Ngapo et al., 2007; Font-i-Furnols and Guerrero, 2014). Poor pork chop quality negatively impacts the consumers’ perception of a product and decreases their likelihood of repurchasing that product (Moeller et al., 2010). Therefore, it is necessary to understand the factors contributing to the ability of postmortem muscle structures and proteins to retain water to improve the consumer eating experience.

Water is held in muscle within and between myofibrils and muscle bundles. Variations in the rate and extent of pH decline (Bowker et al., 2000), degradation of intermediate filament proteins (Melody et al., 2004; Huff-Lonergan and Lonergan, 2005; Zhang et al., 2006), changes in protein solubility (Joo et al., 1999) and protein oxidation (Lund et al., 2007; Liu et al., 2010) are known to cause variation in the water-holding capacity of fresh meat. Low ultimate pH of aged pork loins resulted in more purge loss and tougher product (Zuber et al., 2021), and tougher pork chops had more purge loss (Schulte et al., 2020). The pork industry has also identified genetic mutations to numerous genes, including the halothane (Fujii et al., 1991), melanocortin-4 receptor (Kim et al., 2000), and RN- (Ciobanu et al., 2001) genes, that are either fatal or detrimental to meat quality. The halothane gene impairs the normal function of the ryanodine receptor calcium channels, resulting in rapid muscle contraction and muscle metabolism, and accelerated pH decline that produces an undesirable pale, soft, and exudative pork product. While this detrimental condition has been virtually eliminated in the U.S. commercial pork industry through genetic selection, it is an example of a recent and detrimental condition that negatively impacted the fresh pork industry (Barbut et al., 2008).

Water-holding capacity is influenced by numerous antemortem and postmortem factors (Huff-Lonergan and Lonergan, 2005). Progress has been made in improving pork water-holding capacity; however, several recent studies have documented considerable variation in pork water-holding capacity ranging from 0% to 10% purge loss (Moeller et al., 2010) and more commonly ranging from 0% to 5% purge loss (Boler et al., 2010; di Luca et al., 2016; Carlson et al., 2017b; Schulte et al. 2019, 2020). Liquid chromatography and tandem mass spectrometry (LC–MS/MS) has allowed for the identification and quantification of numerous peptides and proteins within a single sample, which, when coupled with the multiplexing capabilities of isobaric labeling, has increased the throughput of these technologies. The application of LC–MS/MS technologies within meat quality research on a large sample size has been limited. Therefore, the objective of this study was to use tandem mass tag (TMT) analysis to evaluate the aged sarcoplasmic proteome of pork loins classified by purge loss. Previous studies have linked proteolysis to better water-holding capacity (Kristensen and Purslow, 2001; Melody et al., 2004; Huff-Lonergan and Lonergan, 2005) and indicated that proteolysis made some desmin degradation products soluble in the aged sarcoplasmic proteome (Carlson et al., 2017a). It was hypothesized that pork chops with low purge loss would have a greater abundance of structural and intermediate filament proteins in the aged sarcoplasmic proteome, indicating their ability to retain more water.

## Materials and Methods

### Pork loin quality and sensory data collection

The current study was conducted only on pork loins collected from a commercial pork harvest facility following standard humane slaughter practices according to USDA guidelines; therefore, Institutional Animal Care and Use approval was not sought. Pork loins (*N* = 100) were collected from a commercial harvest facility on 1 d postmortem on three separate collection days over 3 wk. The pH measurements were done during the collection using a Hanna HI 9025 pH meter (Hanna Instruments, Woonsocket, RI). Loins were vacuum packaged and transported to Iowa State University on ice. After aging for 12 or 14 d at 4 °C, the loins were removed from the packaging, weighed, and the percent purge was weighed and calculated. Aging time varied to avoid freezing samples before sensory (Carlson et al., 2017b). The pH of the aged samples was measured using a Hanna HI 9025 pH meter (Hanna Instruments) at the blade, the center, and the sirloin portions of the loin, and the average pH across all three locations was calculated. The pH calibration was maintained between each sample to be within a range of pH 6.95 and 7.05. Chops were individually vacuum packaged and stored at 4 °C for 1 d to simulate retail storage. After 1 d, chops were removed from the packaging, the amount of purge was weighed, and the percent purge was calculated.

Loins were cut into 2.54 cm chops and allowed to bloom for 20 min. Visual color scores were applied using a 6-point scale (1 = pale pinkish gray/white; 6 = dark purplish red; National Pork Board, 2000), and visual marbling scores were applied using a 10-point scale (1 = 1% intramuscular fat; 10 = 10% intramuscular fat; National Pork Board, 2000) to chops from the 10th to 12th rib section by trained evaluators using reference cards as a standard. Hunter L* was measured on one chop from the 10th to 12th rib section of each loin using a Minolta Chroma Meter with a D65 light source, 50-mm aperture, and 0° observer.

Two chops were cooked to an internal temperature of 68 °C using a Ninja Foodi Grill (AG302, SharkNinja Operating LLC, Needham, MA) and used to determine chop cook loss and for sensory evaluation. Cooked chops were cut, and 1.5-cm cubes were delivered immediately to each panelist for sensory evaluation. A trained panel (*N* = 4; Institutional Review Board identification number: 14-553-00) evaluated tenderness, chewiness, juiciness, pork flavor, and off-flavor using a 10-point category scale (Carlson et al., 2017b). Lower numbers indicated a lower degree of the given attribute, while higher numbers indicated a higher degree of the given attribute in a sample. An Instron (Instron Industrial Products, Grove City, PA) with a five-point star probe attachment was used for instrumental tenderness measurements, where three compressions were made across each chop and averaged for a final star probe value (Schulte et al., 2019).

Moisture content was determined using the CEM SMART 6 system (Official Method 2008.06, AOAC International), and fat content was determined using the CEM ORACLE system (Official Method 2008.06, AOAC International; CEM Corporation, Matthews, NC, USA). All analyses were done in triplicate and averaged.

### Protein extraction

Frozen pork loin (frozen after aging 12 or 14 d postmortem) containing only the *longissimus dorsi* muscle (~200 g) was homogenized and uniformly powdered in liquid nitrogen. Powdered samples were stored at −80 °C until protein extraction. Proteins soluble under low-ionic conditions were extracted according to Carlson et al. (2017a) with minor modifications. Approximately 1.5 g of powdered sample was homogenized with 4.5 mL of ice-cold low-ionic strength buffer [50 mM Tris–HCl (pH 8.5) and 1 mM ethylenediaminetetraacetic acid (EDTA)] using a Polytron PT 3100 (Polytron, Lucerne, Switzerland). Samples were homogenized in two 10-s bursts and kept on ice. Homogenates were centrifugated at 24,446 × *g* for 30 min at 4 °C, and the supernatant was collected and filtered through cheesecloth. The protein concentration of the filtered supernatant was determined using a Bradford Assay (Bio-Rad Laboratories, Hercules, CA). All samples were adjusted to 10 mg/mL with ice-cold low-ionic strength buffer [50 mM Tris-HCl (pH 8.5) and 1 mM EDTA], vortexed, and stored at −80°C. Separately, all samples were adjusted to 6.4 mg/mL with ice-cold low-ionic strength buffer and further diluted to 4 mg/mL with 0.5 vol of Protein Denaturing Buffer [3 mM EDTA, 3% (wt/vol) sodium dodecyl sulfate (SDS), 30% (vol/vol) glycerol, 0.001% (wt/vol) pyronin Y, and 30 mM Tris–HCl (pH 8.0)] and 0.1 vol of 2-mercaptoethanol. Samples were vortexed, heated on a dry heat block for 15 min at approximately 50 °C, and stored at −80°C.

Confirmation of equal protein concentrations between samples was evaluated by loading 40 µg of each sample onto 15% polyacrylamide resolving gels [10 cm × 10 cm; acrylamide: *N*,*N*ʹ-bis-methylene acrylamide = 100:1 (wt/wt), 0.1% (wt/vol) SDS, 0.05% (vol/vol) *N*,*N*,*N*ʹ,*N*ʹ-tetramethylenediamine, 0.05% (wt/vol) ammonium persulfate, and 0.5 M Tris–HCl (pH 8.8)] overlayed with a 5% polyacrylamide stacking gel [acrylamide: *N*,*N*ʹ-bis-methylene acrylamide = 100:1 (wt/wt), 0.1% (wt/vol) SDS, 0.125% (vol/vol) *N*ʹ*N*ʹ*N*ʹ*N* ʹ-tetramethylenediamine, 0.075% (wt/vol) ammonium persulfate, and 0.125 M Tris–HCl (pH 6.8)] with 10 lanes. Lane 1 of each gel was loaded with Precision Plus Protein All Blue molecular weight standards (Bio-Rad). The remaining lanes were filled with the prepared 4 mg/mL samples, and proteins were fractionated at a constant voltage of 120 V for about 360 V/h with Hoefer 260 Mighty Small II units (Hoefer, Inc., Holliston, MA). The running buffer consisted of 25 mM Tris, 192 mM glycine, 2 mM EDTA, and 0.1% (wt/vol) SDS. Following electrophoresis, gels were stained with Colloidal Coomassie Blue stain [17% (wt/vol) ammonium sulfate, 33% (vol/vol) methanol, 0.03% (vol/vol) phosphoric acid, and 0.1% (wt/vol) Coomassie G-250]. Gels were destained with double-distilled water and changed two times. Gels were imaged with a ChemiImager 5500 (Alpha Innotech Corp.) and analyzed with Alpha Ease FC software (version 3.03, Alpha Innotech Corp.). Samples were again extracted from the tissue level if the total protein within a lane differed from the remaining samples within a gel.

### Tandem mass tag analysis

The 100 commercial pork loins were ranked based on the percent chop purge loss and were classified into three groups: Low (*n* = 27, average purge = 0.33%), Intermediate (*n* = 27, average purge = 0.72%), or High (*n* = 27, average purge = 1.19%) chop purge groups. The low-ionic extract from each pork loin (*N* = 100) was submitted to the Iowa State Protein Facility for TMT analysis; however, only the data from the subset of pork loins ranked on percent chop purge loss (*N* = 81) were used for the analysis. A Master Control sample was generated where 5 µg from each sample was pooled, reduced, alkylated, and digested with trypsin according to the manufacturer’s directions. The Master Control sample was desalted using a C18 MicroSpin Column (SEM SS18V, Nest Group, Inc, Ipswich, MA) and dried using a SpeedVac. The Master Control was reconstituted in 100 mM triethyl ammonium bicarbonate (TEAB), and the concentration of the peptides was determined using a Pierce Colorimetric kit (ThermoFisher). The TMT11-131C reagent (A37724, Thermo Scientific) was reconstituted, and 100 µg of the Master Control was incubated with 0.8 mg of the TMT11-131C reagent for 1 h. The reaction was quenched by adding 8 µL of 5% hydroxylamine in 100 mM TEAB for 15 min.

Each sample (25 µg) was reduced, alkylated, and digested with trypsin according to the manufacturer’s directions. Trypsin digestion was quenched with formic acid, and samples were dried using a Savant SpeedVac Plus (Thermo Scientific). Samples were reconstituted in 40 µL of 5% acetonitrile and 0.1% trifluoroacetic acid and desalted using MicroSpin Columns with C18 silica (SEM SS18V, Nest Group, Inc.). The samples were dried with a SpeedVac and reconstituted in 100 µL of 100 mM TEAB. The peptide concentration was quantified using a Pierce Colorimetric kit (Thermo Scientific). Each sample (25 µg) was incubated with 0.2 mg of a TMT10plex Label Reagents (90110, Thermo Scientific) for 1 h. The reaction was quenched by adding 8 µL of 5% hydroxylamine in 100mM TEAB for 15 min. The samples (*N* = 100) were randomly assigned to 10 different runs, with 10 samples per run. Within a run, samples were randomly assigned to one of the TMT10plex Label Reagents (90110, Thermo Scientific). The digested and labeled peptides from each sample (3 µg) within a run and the master control (3 µg) were pooled (33 µg total) and dried using a SpeedVac. The pooled runs were reconstituted with 33 µL of 5% acetonitrile and 0.1% trifluoroacetic acid.

Chromatographic separation of peptides was achieved using an EASY-nLC 1200 (Thermo Scientific) system with an integrated autosampler. The column consisted of a 75 µm by 20 cm pulled glass emitter (160-2644-5, Agilent Technologies, Santa Clara, CA), where the tip was packed with 5 µm SB-C18 Zorbax packing material (820966-922, Agilent Technologies) and the remainder of the column was packed with UChrom 3 µm packing material (80002, nanoLCMS Solutions, LLC, Rancho Cordova, CA). The column was equilibrated with Buffer A (0.1% formic acid in water). Peptides were eluted and introduced into the Q Exactive Hybrid Quadrupole-Orbitrap Mass Spectrometer (Thermo Scientific) with a Higher Energy Collisional Dissociation (HCD) cell with a Nanospray Flex ion source (Thermo Scientific). The elution gradient consisted of a linear gradient of 0% to 35% Buffer B (0.1% formic acid in acetonitrile) over 240 min, a linear gradient of 35% to 70% Buffer B over 20 min, and a linear gradient of 70% to 100% Buffer B over 4 min at a flow rate of 300 nL/min. Samples were analyzed during MS1 with an automatic gain control (AGC) target of 1 × 10^6^ and a maximum injection time of 80 ms. The MS1 scans were analyzed at 70,000 resolving power, and precursor ions were selected within a scan range of 400 to 2,000 *m/z* during positive ionization mode. The MS2 scans were collected with an isolation window of 1.2 *m*/*z* and fragmented at a 32% normalized collision energy. The ions were analyzed at 35,000 resolving power with an AGC target of 1 × 10^5^ and a maximum injection time of 50 ms.

### Data processing

The data were analyzed using Proteome Discoverer (version 2.5.0.400; Thermo Scientific). Spectra files were searched using Sequest HT and Mascot against a *Sus scrofa* UniProt database. Search parameters of peptide datasets included a 10 ppm precursor mass tolerance and a 0.02 Da fragment mass tolerance for b and y ions produced by HCD fragmentation. Data were searched with the static carbamidomethyl modification of cysteine residues, static TMT-6plex modifications of peptide N-termini and lysine residues, dynamic methionine oxidation, and dynamic deamination of asparagines, and glutamine residues. Identified peptides were filtered to a 1% false discovery rate (FDR). The peptides were then matched to proteins and were filtered to a 1% FDR on the protein level. Proteins were grouped using the maximum parsimony principle from all retained PSM using the default settings of Proteome Discoverer. Protein groups with no unique peptides were removed, and the ambiguity of spectra with more than one PSM was resolved by matching the PSM with the best-fitting protein group and rejecting the other PSM. The best-fitting protein group was the protein group with the highest number of unambiguous PSM and the most unique peptides (Thermo Fisher Scientific, 2020).

### Statistical analysis

The reporter ion intensities were normalized to the total ion count within each run. The ratio of each TMT10-plex reporter ion intensities relative to the TMT11-131C Master control within a run for an identified protein was calculated. Only proteins identified in at least half of the samples (*N* > 40) from the chop purge groups containing at least two unique peptides were included in subsequent analysis. The ratios were Log2 transformed and normalized to the median value within a sample. Statistical analysis was conducted using RStudio (v. 4.1.1) using the *limma* package (Ritchie et al., 2015), where moderated *t* tests were used to make pairwise comparisons between chop purge groups. The Benjamini–Hochberg adjustment method was used at 5% to control the FDR of differentially abundant proteins between chop purge groups. Proteins were considered differentially abundant at an adjusted *P* < 0.05.

Pork quality and sensory attributes were analyzed using RStudio. Summary statistics of each attribute were calculated and reported in Table 1. A one-way analysis of variance was used with the fixed effect of chop purge group. Estimated marginal means were computed using the emmeans function to make pairwise comparisons between purge groups. The fixed effects of gender, harvest date, and sireline were tested separately for each attribute and were reported in Table 2. Significance was denoted by a *P* < 0.05.

**Table 1.** Summary of pork quality traits from the initial 100 (*N* = 100) commercial pork loins

Attribute	Range	Mean	Standard deviation
Chop Purge, %^1^	0.06 to 1.86	0.74	0.37
Loin Purge, %^2^	0.0 to 2.73	0.62	0.59
Cook Loss, %^3^	15.57 to 27.74	22.48	2.55
Moisture Content, %^4^	72.97 to 78.10	74.75	0.88
Lipid Content, %^5^	0.83 to 4.86	2.05	0.78
Marbling Score^6^	1.0 to 4.0	2.1	0.6
Color Score^7^	2.0 to 4.5	3.2	0.6
Aged L*^8^	42.3 to 54.1	48.2	2.3
24 h pH^9^	5.51 to 6.01	5.68	0.11
Aged pH^10^	5.62 to 6.07	5.78	0.10
Star Probe, kg^11^	3.43 to 7.41	5.16	0.82
Tenderness^12^	4.0 to 10.0	6.9	1.1
Chewiness^12^	1.0 to 6.0	3.2	0.9
Juiciness^12^	5.0 to 9.0	6.6	1.0
Flavor^12^	2.0 to 8.0	4.5	1.2
Off Flavor^12^	1.0 to 5.0	1.7	0.8

^1^Percent chop purge = [weight of package with purge (chop removed)/ weight of packaged chop] × 100.

^2^Percent loin purge = [weight of package with purge (loin removed)/ weight of packaged loin] × 100.

^3^Chops were cooked to an internal temperature of 68 °C on Ninja Foodi Grills. Percent cook loss = [(raw weight − cooked weight)/raw weight] × 100.

^4^Moisture content determined using the CEM SMART 6 system (Official Method 2008.06, AOAC International).

^5^Fat content was determined using the CEM ORACLE system (Official Method 2008.06, AOAC International).

^6^National Pork Board standards, 10-point scale (1 = 1% intramuscular fat; 10 = 10% intramuscular fat).

^7^ National Pork Board standards, 6-point scale (1 = pale pinkish gray/white; 6 = dark purplish red).

^8^Hunter L* determined with Minolta Chroma Meter with D65 light source, 50 mm aperture, and 0° observer.

^9^ pH 24 h postmortem taken from the center portion of the *longissimus dorsi*.

^10^Average pH from the blade, center, and sirloin portion of the l*ongissimus dorsi* after aging.

^11^A 5-point star probe attachment fitted with an Instron was used to assess the force needed to compress a chop to 20% of its original height (Carlson et al., 2017b).

^12^As determined by a trained panel (*N* = 4) using a 10-point category scale.

**Table 2.** Summary of pork loin quality characteristics from samples selected as Low, Intermediate, and High based on chop purge loss

Attribute	Low (*n* = 27)	Intermediate (*n* = 27)	High (*n* = 27)	SEM	Purge Class *P*-value	Gender *P*-value	Sireline *P*-value	Harvest date *P*-value
Chop Purge, %^1^	0.33^c^	0.72^b^	1.19^a^	0.03	**<0.01**	0.39	**<0.01**	0.13
Loin Purge, %^2^	0.32^b^	0.87^a^	0.65^a,b^	0.11	**<0.01**	0.25	0.19	0.37
Cook Loss, %^3^	21.0^b^	22.6^a^	23.1^a^	0.48	**<0.01**	0.44	0.38	0.23
Moisture Content, %^4^	74.68	74.65	74.96	0.18	0.39	0.10	0.07	0.08
Lipid Content, %^5^	2.34	2.00	1.90	0.15	0.11	**<0.01**	**<0.01**	0.35
Marbling Score^6^	2.4^a^	2.1^a,b^	1.8^b^	0.1	**<0.01**	**<0.01**	**<0.01**	0.56
Color Score^7^	3.7^a^	3.0^b^	3.0^b^	0.1	**<0.01**	**<0.01**	**0.02**	0.90
Aged L*^8^	47.6^b^	49.5^a^	49.7^a^	0.4	**<0.01**	0.42	**0.04**	0.19
24 h pH^9^	5.77^a^	5.67^b^	5.63^b^	0.02	**<0.01**	**0.03**	**<0.01**	0.64
Aged pH^10^	5.89^a^	5.79^b^	5.74^b^	0.02	**<0.01**	**<0.01**	**<0.01**	0.24
Star Probe, kg^11^	4.68^b^	5.34^a^	5.25^a^	0.15	**<0.01**	**<0.01**	**0.04**	**0.04**
Tenderness^12^	7.5^a^	6.6^b^	6.9^a,b^	0.2	**<0.01**	**0.04**	0.66	0.25
Chewiness^12^	2.8^b^	3.3^a,b^	3.4^a^	0.2	**0.02**	0.71	0.18	0.52
Juiciness^12^	6.8	6.3	6.7	0.2	0.06	0.11	0.68	**0.01**
Flavor^12^	5.3^a^	4.3^b^	4.2^b^	0.2	**<0.01**	0.06	**<0.01**	0.81
Off Flavor^12^	1.3^b^	1.8^a^	1.8^a^	0.1	**0.01**	**0.03**	**0.02**	0.13

^1^Percent chop purge = [weight of package with purge (chop removed)/ weight of packaged chop] × 100.

^2^Percent loin purge = [weight of package with purge (loin removed)/ weight of packaged loin] × 100.

^3^Chops were cooked to an internal temperature of 68 °C on Ninja Foodi Grills. Percent cook loss = [(raw weight − cooked weight)/raw weight] × 100.

^4^Moisture content determined using the CEM SMART 6 system (Official Method 2008.06, AOAC International).

^5^Fat content was determined using the CEM ORACLE system (Official Method 2008.06, AOAC International).

^6^National Pork Board standards, 10-point scale (1 = 1% intramuscular fat; 10 = 10% intramuscular fat).

^7^ National Pork Board standards, 6-point scale (1 = pale pinkish gray/white; 6 = dark purplish red).

^8^Hunter L* determined with Minolta Chroma Meter with D65 light source, 50 mm aperture, and 0° observer.

^9^ pH 24 h postmortem taken from the center portion of the *longissimus dorsi*.

^10^Average pH from the blade, center, and sirloin portion of the l*ongissimus dorsi* after aging.

^11^A 5-point star probe attachment fitted with an Instron was used to assess the force needed to compress a chop to 20% of its original height (Carlson et al., 2017b).

^12^As determined by a trained panel (*N* = 4) using a 10-point category scale.

^a,b,c^Means within rows with different superscripts are significantly different (*P* < 0.05).

## Results and Discussion

The aged, fresh loin composition, quality, and sensory attributes are summarized in Table 1, with the range, mean, and standard deviation reported for the total population of pork chops (*N* = 100). Table 2 reports the estimated marginal means of pork chops (*N* = 81) classified as Low, Intermediate, and High based on chop purge loss. There was a 0.86% difference in chop purge between the Low and High purge groups. The percent loin purge was greater in the Intermediate vs. the Low group (*P* < 0.05) but not between the Low and High groups (*P* > 0.05). Cook loss was less (*P* < 0.05) in the Low vs. the Intermediate and High groups. Moisture and lipid content were not different (*P* > 0.05) between purge groups. The Low purge group had higher visual marbling scores (*P* < 0.05) than the High purge and higher visual color scores (darker purplish red; *P* < 0.05) than the Intermediate and High groups. The Low purge group was the darkest with the lowest L* value (*P* < 0.05) and had a greater pH at 24 h (*P* < 0.05) and after aging (*P* < 0.05) than the Intermediate and High groups. The Low purge group had a lower star probe value (*P* < 0.05) than the Intermediate and High purge groups. The trained sensory panel rated the Low purge group as more tender than the Intermediate purge group, as evidenced by a greater tenderness score (*P* < 0.05). The Low purge group was less chewy (*P* < 0.05) than the High purge group and had more pork flavor (*P* < 0.05) and less off flavor (*P* < 0.05) than the Intermediate and High purge groups. There was no difference in sensory panel juiciness scores between purge groups (*P* > 0.05). The Low purge group was generally of greater quality than the Intermediate or High purge groups as evidenced by a higher color score and a lower Hunter L* value, a lower star probe value, a higher sensory panel tenderness and flavor score, and a lower sensory panel chewiness and off-flavor score. The Low purge group had a greater pH (approximately 0.15 pH units) at 24 h and 14 d postmortem, which could be contributing to the quality differences between the purge groups. Previous studies have shown that postmortem pH can influence pork quality attributes (Boler et al., 2010; Zuber et al., 2021)

After spectral and peptide matching, 807 protein groups (refer to Data Processing for information on protein grouping) were identified in at least 1 of the 10 LC–MS/MS runs. Proteins containing at least two unique peptides and present in at least half of the samples (*N* > 40) were included in the analysis, resulting in a total of 438 proteins. No ­differences were observed between the Low and Intermediate or between the Intermediate and High purge groups. There were 40 proteins differentially abundant between the Low and High purge groups. Table 3 summarizes the differentially abundant proteins between the Low and High purge groups. The FASTA sequence of proteins initially labeled “Uncharacterized Protein” was searched using the BLAST feature of UniProt (Altschul et al., 1997). The resulting protein with the lowest expect value was used to rename the protein, and the changes are noted in Table 3. The differentially abundant proteins could be classified primarily as involved in structure or contraction, calcium regulation, sarcoplasmic reticulum-associated, chaperone or heat shock proteins, metabolic, LIM domain-containing, and others.

**Table 3.** Summary of proteins in the aged sarcoplasmic proteome that were differentially abundant between Low and High purge groups

Protein description	Accession number^1^	Subcellular location	Sequence coverage^2^	Unique peptides	Log_2_ fold change^3^	Adjusted *P*-value
*Structural*						
Titin^4^	A0A5G2QM05	Thick filament	61	27	0.528	0.026
Desmin	P02540	Intermediate filament	52	22	0.527	0.005
Filamin C	F1SMN5	Z-line	31	56	0.338	0.005
Obscurin^4^	A0A5G2QZ79	M-line	21	10	0.255	0.010
Dystrophin	Q5GN48	Costamere	11	30	−0.182	0.036
Nebulin	F1SHX0	Thin filament	25	4	−0.244	0.005
*Contractile*						
Troponin C, skeletal muscle (TnC)	P02587	Thin filament	60	9	0.469	0.025
Myosin regulatory light chain 2^4^ (RLC-2)	A0A4X1TZM9	Thick filament	58	10	0.233	0.036
*Sarcoplasmic reticulum*						
Calcium transport ATPase 1 (SERCA-1)	A0A5G2R940	SR^5^	43	24	0.499	0.022
Calcium transport ATPase 2 (SERCA-2)	A0A4X1U5D4	SR^5^	19	6	0.438	0.010
Calsequestrin 1	F1RJW7	SR^5^	30	8	0.766	0.012
Sarcalumenin^4^	A0A4X1VRP4	SR^5^	26	17	0.545	0.014
Junctophilin 1	F1RWK0	SR^5^membrane	17	9	−0.197	0.036
*Chaperone Proteins*						
CCT-theta	A0A480X895	Multiple	21	9	0.356	0.013
Heat shock protein beta 1^4^ (HSPB1)	A0A5S6G3Y8	Multiple	16	4	0.405	0.013
Heat shock protein beta 5^4^ (HSPB5)	A0A4X1TDZ5	Multiple	27	4	0.702	0.005
Heat shock protein beta 8^4^ (HSPB8)	A0A286ZID4	Multiple	10	2	0.485	0.005
*Antioxidants*						
Maillard deglycase	Q0R678	Sarcoplasm	80	15	−0.160	0.016
*Metabolic*						
ATP dependent 6-phosphofructokinase	A0A287B8N1	Sarcoplasm	47	29	0.380	0.005
Fructose bisphosphate aldolase	A0A4X1U0N5	Sarcoplasm	79	3	−0.153	0.005
Beta enolase^4^	A0A4X1USV7	Sarcoplasm	67	20	−0.167	0.022
AMP deaminase 1	A6NA29	Sarcoplasm	33	22	0.495	0.012
Isocitrate dehydrogenase [NADP], mitochondrial (Fragment)	P33198	Mitochondrial Matrix	36	13	0.423	0.005
Succinate--CoA ligase [ADP/GDP-forming] subunit alpha, mitochondrial	A0A5G2R4W7	Mitochondrial Matrix	7	2	0.424	0.025
Fumarate hydratase, mitochondrial	A0A4X1THY9	Mitochondrial Matrix	30	11	0.521	0.010
Phosphoinositide phospholipase C	A0A4X1UIS9	Sarcoplasm	12	7	0.314	0.041
*LIM Domain*						
Four and a half LIM domains protein 1 ­isoform 5 (FHL1)	F6PXR6	Z-line	60	16	0.499	0.005
LIM and cysteine-rich domains protein 1 (LMCD1)	A0A5G2Q8A7	Z-line	28	8	0.212	0.040
LIM domain-binding protein 3^4^ (LDB3)	A0A287A435	Z-line	47	15	−0.235	0.010
*Uncategorized*						
14_3_3 protein zeta/delta	A0A480PLY3	Sarcoplasm	34	4	0.743	0.022
14_3_3 protein epsilon^4^	A0A5G2QSR7	Sarcoplasm	40	8	0.469	0.014
14_3_3 protein gamma^4^	F2Z4Z1	Sarcoplasm	54	8	0.487	0.013
CXXC motif containing zinc-binding protein	F1S765		36	5	−0.202	0.027
Leukotriene A(4) hydrolase	A0A480LQK0		29	14	0.286	0.005
MTH 938 domain-containing protein^4^	A0A4X1U924	Sarcoplasm	47	6	−0.211	0.005
Obg-like ATPase 1	A0A4X1UNV8		43	14	0.172	0.037
RCSD domain containing 1	A0A286ZSH0		8	2	-0.720	0.022
Reticulon	Q6IM77		12	3	0.451	0.048
RRM domain-containing protein	A0A4X1WBH3		4	2	−0.276	0.032
SH3 domain-binding glutamic acid-rich protein isoform a (Fragment)	A0A480TH91		37	4	−0.281	0.022

^1^Accession Number = Uniprot Accession Number.

^2^Sequence Coverage = Percent of the total number of identified amino acids/total number of amino acids.

^3^Log_2_ Fold Change = Low/High chop purge group; Positive number = Greater in Low vs. High chop purge group, Negative number = Greater in High vs. Low chop purge group.

^4^These proteins were initially labeled “Uncharacterized” or were labeled with a less commonly identified name. The FASTA sequence of the protein was used to match each protein to a more commonly recognized or accepted name using the UniProt BLAST feature.

^5^SR, sarcoplasmic reticulum.

### Structural and Contractile Proteins

There was a greater abundance of titin (~3,000 kDa), obscurin (~720 kDa), filamin C (~290 kDa), desmin (~55 kDa), troponin C (18 kDa), and myosin regulatory light chain 2 (19 kDa) in the aged sarcoplasmic proteome of the Low vs. High purge group. Nebulin (~600 to 900 kDa) and dystrophin (427 kDa) were greater in the aged sarcoplasmic proteome of the High vs. Low purge group. In their intact form, these proteins are not soluble under low-ionic conditions and would require greater ionic conditions or detergents to be soluble; thus, their identification in the low-ionic strength soluble fraction would not be expected in early postmortem pork. Their presence in the aged sarcoplasmic proteome indicates the degradation of these proteins. The cleavage or degradation of these intact proteins can create degradation products, which could result in soluble fragments under the extraction conditions used (Carlson et al., 2017a). The bottom-up proteomics approach would make it nearly impossible to determine if these tryptic peptides originated from an intact protein or a degradation product. Because the intact proteins would be insoluble under low-ionic strength conditions, it could be reasonably inferred that these identified peptides and proteins are degradation products. The fractionation of these proteins based on solubility is crucial to this analysis and provides unique insight into the aged sarcoplasmic proteome that could not be achieved without fractionation.

Titin is the largest protein in mammalian tissue and spans half the length of the sarcomere from the Z-line to the M-line. Desmin is an intermediate filament that connects adjacent myofibrils at the periphery of the myofibrillar Z-line and connects myofibrils to other cellular structures (Huff Lonergan et al., 2010). Filamin-C localizes in the Z-line and sarcolemma, crosslinks F-actin, and interacts with numerous other proteins (Mao and Nakamura, 2020). The degradation of titin and desmin has been associated with pork chops with less drip loss (Melody et al., 2004). The abundance of intact titin, filamin, and desmin was greater in pork chops classified as high vs. low star probe, where the high star probe group had more cook loss and was rated less juicy by a trained sensory panel (Carlson et al., 2017b). Desmin (Schulte et al., 2020), titin, and filamin-C (Huff-Lonergan et al., 1996; Carlson et al., 2017b) have been indicators of postmortem proteolysis and tenderness in pork and beef. Cellular environments that promote meat tenderness may also promote water-holding capacity in postmortem skeletal muscle (Huff-Lonergan and Lonergan, 2005).

Obscurin binds to titin and small ankyrin 1 to connect the cytoskeleton to the sarcoplasmic reticulum (Kontrogianni-Konstantopoulos et al., 2003; Meyer and Wright, 2013). Myosin regulatory light chain 2 (RLC-2) is located near the neck region of the myosin head on the thick filament. RLC-2 modulates skeletal muscle contraction through phosphorylation sites, thereby increasing calcium sensitivity and contractile force (Sitbon et al., 2020). Troponin-C (TnC) is the calcium-binding component of the troponin complex. Upon calcium binding to TnC, a conformational shift alters TnC’s interaction with troponin-T (TnT) and troponin-I (TnI), ultimately allowing for the interaction and binding of actin and myosin during skeletal muscle contraction (Zot and Potter, 1987). A greater abundance of obscurin, RLC-2, and TnC was observed in the aged sarcoplasmic proteome of the Low vs. High purge group. Few studies have quantified and related these proteins to meat quality. Obscurin abundance in the low-ionic fraction was greater in bison *longissimus lumborum* (LL) vs. *psoas major* (PM) on 2 d postmortem (Hasan et al., 2022), while in the total protein fraction, obscurin was less abundant in pork LL with high centrifugal drip loss vs. low drip loss immediately after exsanguination (Zequan et al., 2021). Schulte et al. (2020) showed a greater abundance of one spot of RLC-2 on 1 d postmortem in the low-ionic extract from chops classified as high vs. low star probe, where these star probe groups differed in purge loss on 14 and 21 d postmortem. Four spots of RLC-2 have been identified in pork on 1 d postmortem in the total protein fraction (Hwang et al., 2005; Alessandro et al., 2011). Troponin-C is not a substrate for the calpain family (di Lisa et al., 1995) or cathepsin B proteases (Masanori et al., 1992). While TnC may not be a substrate for these specific proteases, the degradation of TnT or TnI may release TnC from the troponin complex. The release from the troponin complex alone or in combination with degradation by other proteases may alter the solubility of TnC.

Nebulin is a large protein that spans the length of the thin filament, with its C-terminus embedded in the Z-line and extending to the ends of the thin filament (Huff Lonergan et al., 2010). Dystrophin connects the cytoskeletal network to the sarcolemma by connecting actin to dystroglycan, and its function is primarily to transmit the contraction force laterally across the cell and facilitate important connections between the cytoskeleton, sarcolemma, and extracellular matrix (Allen et al., 2016). In the current study, there was a greater abundance of nebulin and dystrophin in the aged sarcoplasmic proteome of the High compared to the Low purge group. Previous studies have shown that the abundance of intact dystrophin was less at 24 and 94 h postmortem in the total protein fraction of pork *LL* samples with greater drip loss than *PM* samples (Wojtysiak, 2020). Conversely, in turkey (Wojtysiak and Górska, 2018) and poultry (Wojtysiak et al., 2019) *pectoralis major* muscles, the abundance of intact dystrophin was greater in high drip loss samples at 24 and 48 h postmortem. Nebulin degradation has been associated with postmortem proteolysis and meat tenderization (Melody et al., 2004; Huff Lonergan et al., 2010). The presence of nebulin and dystrophin in the low-ionic fraction is associated with greater purge loss. Nebulin (Huff-Lonergan et al., 1996) and dystrophin (Cottin et al., 1992) are substrates for the calpain family of proteases. The direction of the fold change of these observations is inverse of the other ­differentially abundant structural and contractile proteins. The rapid degradation of nebulin and dystrophin could indicate pork chops with poor water-holding capacity.

### Calcium-regulating, sarcoplasmic reticulum, and sarcoplasmic reticulum associated proteins

Based on chop purge loss classification, five proteins—calsequestrin-1, sarco/endoplasmic reticulum ATPases (SERCA)-1, SERCA-2, junctophilin, and sarcalumenin—were differentially abundant. Calsequestrin-1, SERCA-1, SERCA-2, and sarcalumenin were all greater in abundance in the Low vs. High purge group. Junctophilin was greater in abundance in the High vs. Low purge group. Junctophilin is key in maintaining the architectural connection between the sarcoplasmic reticulum (SR) and the transverse tubule; however, junctophilin is not specifically a component in the SR but instead is located outside the SR (Landstrom et al., 2014; Lehnart and Wehrens, 2022).

Calcium release and sequestration are critical mechanisms to regulate muscle contraction and relaxation in living muscles. Calcium also serves to regulate proteases, enzymes, and cellular metabolism. The SR is a muscle cell organelle that controls calcium availability in the sarcoplasm. After sarcolemma depolarization, a conformational change in the dihydropyridine receptor allows interaction with the ryanodine receptor, which results in the release of calcium from the terminal cisternae of the SR (Rossi et al., 2022). Calcium uptake from the sarcoplasm into the SR lumen is coordinated primarily by SERCA proteins. Calsequestrin (Rossi et al., 2022) and sarcalumenin (Yoshida et al., 2005) are calcium-binding proteins in the SR lumen critical to calcium sequestration.

It is reasonable to propose that disruption of the SR membrane would increase the abundance of these proteins in the aged sarcoplasmic proteome. The SR vesicle has been previously isolated using differential centrifugation (Meissner, 1975), and under the conditions of the current experiment, the SR would be pelleted during centrifugation. Therefore, the greater abundance of calsequestrin-1, SERCA-1, SERCA-2, and sarcalumenin in the aged sarcoplasmic proteome of the Low purge group may indicate a more rapid or extensive disruption of the SR and, ultimately, calcium regulation postmortem.

In a previous study, SERCA-1 abundance in the total protein fraction was less at 1 h postmortem in pork chops with greater drip loss (Wang et al., 2019). The activity of SERCA-1 was also less when standardized on a total protein basis, which the authors attribute to the greater nitrosylation of SERCA-1 in the high drip loss group (Wang et al., 2019). The consideration of post-translational modifications of SERCA remains an important consideration in evaluating the activity of these enzymes. In the total protein fraction of pork LL, the abundance of sarcalumenin increased from 0 to 72 h postmortem (Morzel et al., 2004)

The current study identified the SR-related proteins as differentially abundant aged sarcoplasmic proteome, compared to the total protein fraction from the studies by Morzel et al. (2004) and Wang et al. (2019). This raises the question about the integrity of the SR vesicle since calsequestrin-1, SERCA-1, SERCA-2, and sarcalumenin reside either within the SR vesicle or within the SR membrane. If the SR is disrupted, the factors that induce SR disruption and the postmortem duration in which the SR remains intact are not well characterized. The SR disruption could increase sarcoplasmic calcium concentrations, activating the calpain proteases to promote proteolysis and improve the pork products’ water-holding capacity (Huff-Lonergan and Lonergan, 2005).

### Chaperone proteins

In the current study, four chaperone proteins—heat shock protein (HSP) β-1 (HSPB1), α-crystallin B chain (HSPB5), heat shock protein β-8 (HSPB8), and chaperonin-containing T-complex (CCT)-θ were greater in abundance in the Low vs. High purge group. HSPB1, HSPB5, and HSPB8 are classified as small HSP, a family of heat shock proteins that vary in molecular weight (12 to 42 kDa), share a conserved alpha-crystallin domain, and can form large oligomeric structures (Kampinga et al., 2009). These larger oligomeric protein complexes interact with hundreds of denaturing proteins to prevent their irreversible aggregation in an ATP-independent manner but do not participate in refolding these proteins (Haslbeck and Vierling, 2015; Haslbeck et al., 2019). CCT proteins are large (800 to 900 kDa) double-ring complexes of proteins, each containing eight separate subunits (Saibil, 2013). The CCT protein complex encapsulates proteins up to 60 kDa in an ATP-dependent manner to ensure proper protein folding. Mammalian CCT binds actin, tubulin, and many other proteins to ensure correct protein folding (Vallin and Grantham, 2019).

In living muscle, HSP are highly expressed in response to stress. Heat stress increased the abundance of HSPB1, HSPB5, HSPB6, and HSPA1B in the *semitendinosus* muscle from heat-stressed pigs (Cruzen et al., 2015). Those authors suggested that these proteins may protect cellular components and proteins during periods of stress in pork.

Small HSP have also been previously associated with meat quality. In pork LD classified as high or low pH based on 24 h pH, one spot of HSPB1 and HSPB5 was more abundant in the high compared to the low pH group. The high pH group also had less drip loss than the low pH group (Subramaniyan et al., 2017). The abundance of HSPB5 and HSPB6 decreased during postmortem aging, while a lower molecular weight band of HSPB1 increased during aging (Ma and Kim, 2020). Lomiwes et al. (2014) summarized studies that identified small HSP in postmortem skeletal muscle, where primarily HSPB1 and HSPB5 were related to meat quality traits. From a summary of multiple studies, HSPB1 and HSPB6 abundances were commonly associated with beef tenderness, but the direction of the relationship of HPSB1 and HSPB6 with beef tenderness was not always the same (Picard and Gagaoua, 2020). While highly expressed in skeletal muscle, little is known about the function or mechanisms of HSPB8 (Basha et al., 2012). Few studies have quantified and related CCT protein abundance with meat quality, but some have shown that CCT-α abundance decreases postmortem in pork LL (Morzel et al., 2004) and that CCT-θ abundance was greater in high quality vs. low-quality Korean beef LL (Kim et al., 2008). These previous results support the current study’s observations that pork chops with greater water-holding capacity have a greater abundance of chaperone proteins.

Posttranslational modifications are crucial for many chaperone proteins’ functionality and can alter the oligomeric state and activity of the protein complex (Haslbeck et al., 2019). Multiple spots of small HSP have been identified previously in postmortem skeletal muscle, including six spots of HSPB1 in pork *semitendinosus* (Cruzen et al. 2015, 2017), seven spots of HSPB1 in pork *adductor* (Hollung et al., 2009), three spots of HSPB1 and four spots of HSPB5 in pork *semimembranosus* (Laville et al., 2009), and potential degradation products of HSPB1 in beef (Ma and Kim, 2020) and HSPB5 in pork (Cruzen et al., 2015). These previous studies underscore the posttranslational modifications that small HSP can undergo, and although these modifications are not easily detected or quantified with the methodology utilized in the current experiment, an appreciation for the diverse forms is warranted.

### Metabolic proteins

The Low purge group had a greater abundance of phosphofructokinase-1 and AMP deaminase and a lesser abundance of fructose bisphosphate aldolase and beta-enolase than the High purge group. Phosphofructokinase-1 is a rate-limiting enzyme in anaerobic glycolysis during the conversion of fructose 6-phosphate to fructose 1,6-bisphosphate because its activity decreased as pH declines and was shown to lose all activity in vitro below pH 5.5 (England et al., 2014). Fructose bisphosphate aldolase catalyzes the conversion of fructose 1,6-bisphosphate to dihydroxyacetone phosphate and glyceraldehyde 3-phosphate, while beta-enolase catalyzes the reaction of 2-phosphoglycerate to phosphoenolpyruvate. AMP deaminase catalyzes the conversion of AMP to IMP, and AMP is known to activate phosphofructokinase-1 allosterically.

Isocitrate dehydrogenase, succinyl CoA ligase subunit alpha, and fumarate hydratase were more abundant in the Low vs. High purge group. These proteins are enzymes in the citric acid cycle that coordinate the reduction of electron carriers like NADH and FADH_2_. The citric acid cycle occurs within the mitochondrial matrix. Identifying these proteins in the aged sarcoplasmic proteome may indicate mitochondrial disruption to some degree; however, these data alone are insufficient indications of mitochondrial disruption.

Postmortem skeletal muscle metabolism ultimately contributes to meat quality. It is recognized that metabolite abundance, enzyme abundance, and enzyme activity dictate postmortem muscle metabolism and influence ultimate meat quality (Bowker et al., 2000; Scheffler and Gerrard, 2007). The abundance of phosphofructokinase-1 was greater at 45 min vs. 14 d postmortem, while AMP deaminase was greater at 14 d vs. 45 min postmortem (Zuber et al., 2021). AMP deaminase activity and abundance have previously explained some variations in postmortem pH decline and muscle metabolism (England et al., 2015). Isocitrate dehydrogenase subunit alpha was more abundant in the sarcoplasmic fraction of pork chops classified as high vs. low star probe at 1 d postmortem (Schulte et al., 2020). While the current study provides only a single observation of the aged sarcoplasmic proteome, variation in glycolytic and citric acid cycle enzyme abundance is related to pork water-holding capacity. Few other studies have detected mitochondrial citric acid cycle enzymes in the sarcoplasmic fraction, likely due to the lack of fractionation of muscle proteins based on solubility prior to sample analysis. There is a need to understand the mechanisms regarding the presence of these mitochondrial enzymes in the sarcoplasmic fractions. Also, factors that influence the abundance and activity of the identified glycolytic and citric acid cycle enzymes warrant additional research to better understand pork water-holding capacity.

### LIM domain-containing proteins

The LIM proteins contain one to five domains with 8 highly conserved amino acid residues where residues 1 to 4 and residues 5 to 8 coordinate the binding of one zinc ion each (Kadrmas and Beckerle, 2004). The number and location of LIM domains and other functional domains showcase the diversity of LIM domain proteins. The LIM proteins form critical protein–protein interactions with diverse protein targets ranging from cytoskeletal to nuclear proteins. Li et al. (2012) summarized some LIM domain proteins, alternative protein names, and cellular functions.

Four and a half LIM domains protein 1 (FHL1) and LIM and cysteine-rich domain protein 1 (LMCD1) were greater in abundance in the Low vs. High purge group, while LIM domain binding protein 3 (LDB3) was less abundant in the Low vs. High purge group. FHL1 is highly expressed in skeletal muscle (Morgan and Madgwick, 1999), localizes to the Z-line and M-line in mature muscle cells, and competes with myosin to bind myosin-binding protein C (McGrath et al., 2006). As its name implies, FHL1 contains four LIM domains and a half N-terminus LIM domain. Multiple functions have been ascribed to FHL1, including sarcomere assembly, response to biomechanical stress, and muscle hypertrophy (Shathasivam et al., 2010); however, specific mechanisms of FHL1 in skeletal muscle are poorly characterized. LMCD1 is primarily localized to the Z-line (Li et al., 2012) and is necessary for cardiomyocyte (Frank et al., 2010) and skeletal muscle (Ferreira et al., 2019) hypertrophy. LMCD1 contains two LIM domains, an amino-terminal cysteine-rich domain and a PET domain (Ferreira et al., 2019). LDB3 contains a PDZ domain that associates with α-actinin, localizes to the Z-line (Krcmery et al., 2010), and plays a role in sarcomere integrity (Li et al., 2012).

The LIM domain family of proteins has recently been identified in meat quality studies utilizing proteomic techniques. The association of FHL1 in beef has been more recently studied and related to greater marbling (Bonnet et al., 2020) and negatively associated with Warner–Bratzler shear force (Gagaoua et al., 2018). Boudon et al. (2020) identified FHL1 and LMCD1 as greater in abundance in LL steaks classified as tender vs. tough based on Warner–Bratzler shear force values. A difference in LDB3 abundance between two crossbred cattle breeds has been reported (Keady et al., 2013). Additional research is needed to further define the role of these LIM proteins within skeletal muscle and better characterize their association with meat quality.

## Conclusion

Pork water-holding capacity is a critical quality attribute influencing fresh pork products’ eating quality and value. Over the last few decades, research has identified factors influencing water-holding capacity, including genetic traits, postmortem proteolysis, and the rate and extent of pH decline. However, pork water-holding capacity remains variable, and reliable means of producing fresh pork with consistently high water-holding capacity are not yet identified. These data describe changes in the aged sarcoplasmic proteome of pork loins where the Low purge group had a greater abundance of myofibrillar degradation products, SR and calcium regulating proteins, and chaperone-related proteins. The Low purge group also had a greater abundance of citric acid cycle enzymes, whereas the High purge group tended to have a greater abundance of glycolytic enzymes. These differentially abundant proteins in the aged sarcoplasmic proteome provide supporting evidence to existing observations that the proteolysis of certain myofibrillar proteins is linked to greater water-holding capacity. Future research should evaluate the extent and timing postmortem to which SR disruption and calcium homeostasis occur and the role of chaperone and other proteins identified in this study contribute to meat water-holding capacity.

## Abbreviations

AGC: automatic gain control
EDTA: ethylenediaminetetraacetic acid
FDR: false discovery rate
HCD: higher energy collisional dissociation
LC–MS/MS: liquid chromatography–tandem mass spectrometry
SDS: sodium dodecyl sulfate
SDS–PAGE: sodium dodecyl sulfate–polyacrylamide gel electrophoresis
TEAM: triethyl ammonium bicarbonate
TMT: tandem mass tag
